# Healthcare-Associated Infections Caused by *Mycolicibacterium neoaurum*

**DOI:** 10.3201/eid2908.230007

**Published:** 2023-08

**Authors:** Kate Shapiro, Shane J. Cross, Ted H. Morton, Hiroto Inaba, Ashley Holland, Francisca R. Fasipe, Elisabeth E. Adderson

**Affiliations:** St. Jude Children’s Research Hospital, Memphis, Tennessee, USA (K. Shapiro, S.J. Cross, T.H. Morton, H. Inaba, A. Holland, E.E. Adderson);; University of Tennessee Health Sciences Center, Memphis (S.J. Cross, T.H. Morton, H. Inaba, E.E. Adderson);; Mercy Children’s Hospital, Springfield, Missouri, USA (F.R. Fasipe)

**Keywords:** tuberculosis and other mycobacteria, healthcare-associated infections, bacteremia, *Mycolicibacterium neoaurum*, central line-associated bacteremia, endocarditis, peritonitis, urinary tract infection, skin infection, soft tissue infection, surgical wound infection, pacemaker infection, leukemia, bacteria, zoonoses, Australia, Canada, China, Japan, Italy, South Korea, Spain, United Kingdom, United States

## Abstract

*Mycolicibacterium* *neoaurum* is a rapidly growing mycobacterium and an emerging cause of human infections. *M.* *neoaurum* infections are uncommon but likely underreported, and our understanding of the disease spectrum and optimum management is incomplete. We summarize demographic and clinical characteristics of a case of catheter-related *M.* *neoaurum* bacteremia in a child with leukemia and those of 36 previously reported episodes of *M.* *neoaurum* infection. Most infections occurred in young to middle-aged adults with serious underlying medical conditions and commonly involved medical devices. Overall, infections were not associated with severe illness or death. In contrast to other mycobacteria species, *M.* *neoaurum* was generally susceptible to multiple antimicrobial drugs and responded promptly to treatment, and infections were associated with good outcomes after relatively short therapy duration and device removal. Delays in identification and susceptibility testing were common. We recommend using combination antimicrobial drug therapy and removal of infected devices to eradicate infection.

## Abstract 2

This rapidly growing mycobacterium responded promptly to treatment, but delays in identification and susceptibility testing were common.

Comprehensive phylogenetic and genomic studies support the division of the genus *Mycobacterium* into 5 main clades: the emended genus *Mycobacterium*, *Mycolicibacter* gen. nov, *Mycolicibacillus* gen. nov., *Mycolicibacterium* gen. nov., and *Mycobacteroides* gen. nov (*1*). *Mycolicibacterium* spp. include rapidly growing mycobacteria (RGM) that are not part of the *Mycobacterium abscessus* complex (i.e., *Mycobacteroides* gen. nov., which includes *M. abscessus, M. chelonae*, *M. franklinii*, *M. immunogenum*, and *M. saopaulense*). 

*Mycolicibacterium* spp. are considered to have low pathogenicity, but some are associated with human infection. *Mycolicibacterium neoaurum*, originally described by Tsukamura in 1972, is derived from the Greek word for new gold because of distinctive yellow-orange colonies (*2*). Since its identification, increasing numbers of case reports and small case series of invasive *M. neoaurum* infections have been described. Infections are likely underrecognized because many laboratories do not identify all mycobacteria at the species level. Bacteremia might also be missed or isolates inappropriately dismissed as contaminants because mycobacteria might require longer culture incubation than conventional bacterial pathogens, and they are ubiquitous in the environment. 

Although best practices for treating infections caused by more commonly reported RGM species are now recognized, our understanding of disease spectra and best infection management strategies for rarer RGM species, such as *M. neoaurum*, remains incomplete. Therefore, to improve clinical awareness of *M. neoaurum*, we summarized demographic and clinical characteristics of 36 previously reported episodes and report an additional case of *M. neoaurum* infection.

## Methods

We obtained demographic and clinical characteristics of the patient in the reported case from health information records. We searched PubMed and Embase (https://www.embase.com) databases by using the terms neoaurum, *Mycobacterium neoaurum*, and *Mycolicibacterium neoaurum*. We cited cases that reported individual clinical data and were published in any year or language. Patient characteristics were summarized by using descriptive statistics. Analyses were performed by using Stata version 16.1 software (StataCorp LLC).

## Results

### Case Report

A 21-month-old boy with low-risk B cell acute lymphoblastic leukemia (ALL) in remission was treated by using the St. Jude Children’s Research Hospital TOTAL Therapy Study 17 protocol (ClinicalTrials.gov identifier NCT03117751), which is similar to the low-risk arm of the TOTAL Therapy Study 16 protocol (identifier NCT00549848) (*3*). In brief, remission induction consists of prednisone, vincristine, daunorubicin, and pegylated asparaginase, then cyclophosphamide, cytarabine, and mercaptopurine. Consolidation therapy consists of 4 courses of high-dose methotrexate and mercaptopurine. Continuation therapy consists of 120 weeks of mercaptopurine, dexamethasone, vincristine, and methotrexate interrupted by 2 reinduction cycles with dexamethasone, vincristine, and pegylated asparaginase. Triple intrathecal therapy with methotrexate, dexamethasone, and cytarabine was provided to control leukemia in the central nervous system. 

During week 13 of continuation therapy, the patient had a fever of 103°F, cough, coryza, anorexia, and diarrhea and was hospitalized 1 day after onset of those signs and symptoms. His medical history was remarkable because of episodes of mucositis associated with chemotherapy, a recent respiratory syncytial virus upper respiratory tract infection, and distant placement of a subcutaneous port (SCP) for intravenous access. He received trimethoprim/sulfamethoxazole (TMP/SMX) for *Pneumocystis* pneumonia prophylaxis. He lived with his family in an urban area, and no history of difficulties accessing his SCP or erythema, discharge, or tenderness at the SCP insertion site had been observed. His physical examination was unremarkable except for mild pallor. His blood leukocyte count was 10.9 × 10^3^ cells/μL (reference range 6.0–17.0 × 10^3^ cells/μL), consisting of 77% neutrophils, 9% lymphocytes (absolute lymphocyte count 0.97 × 10^3^ cells/μL [reference range 1.20–4.00 × 10^3^ cells/μL]), and 13% monocytes. He was mildly anemic; hemaglobin level was 10.7 g/dL (reference range 11.3–12.3 g/dL). Serum C-reactive protein was elevated at 10.7 mg/L (reference range <5.0 mg/dL), aspartate aminotransferase level was 136 U/L (reference range 10–50 U/L), and alanine aminotransferase level was 520 U/L (reference range <50 U/L). Serum bilirubin was within reference range. A respiratory PCR panel was positive for respiratory syncytial virus.

The patient’s symptoms resolved overnight, and he was discharged. One bacteria species was isolated after a 5-day incubation of blood cultures obtained from the patient’s SCP at hospital admission by using the BacT/ALERT automated microbial detection system (bioMérieux). The organism was initially reported as a gram-positive coccobacillus but stained weakly, prompting acid-fast bacillus (AFB) staining, which gave positive results. *M. neoaurum* was identified initially by matrix-assisted laser desorption/ionization time-of-flight mass spectrometry and confirmed by 16S rRNA sequencing; both of those analyses were performed at the Mayo Clinic Laboratories (Rochester, MN, USA). The Mayo Clinic Laboratories also performed antimicrobial drug susceptibility testing by using the broth microtiter dilution method. Results were reported as MICs (in μg/mL) and interpreted according to Clinical and Laboratory Standards Institute guidelines (*4*).

The patient was readmitted for further bacteremia evaluation 9 days after initial blood cultures were obtained. *M. neoaurum* was again isolated from blood drawn from the SCP and catheter tip, but blood cultures obtained from a peripheral vein were sterile. Results of chest radiograph were unremarkable. We initiated empirical therapy with intravenous imipenem/cilastatin, oral azithromycin, and oral ciprofloxacin. We removed the SCP on hospital day 3, and 3 blood cultures obtained after port removal were sterile. We inserted a central catheter line peripherally on hospital day 6. Antimicrobial drug susceptibility tests showed that the *M. neoaurum* isolate was susceptible to cefoxitin, imipenem, ciprofloxacin, moxifloxacin, amikacin, tobramycin, doxycycline, TMP/SMX, and linezolid and resistant to clarithromycin. The MIC for tigecycline was 0.12 μg/mL. We ultimately treated the patient with imipenem/cilastatin, azithromycin, and ciprofloxacin for 16 days, then with TMP/SMX and ciprofloxacin for 26 days, and he remained well 15 months later.

### Literature Review

We found 238 articles in the literature and included 31 reports describing 36 cases in this review (Appendix Table). Including the case report we described, the median age of patients was 46 (interquartile range [IQR] 25–59) years, and 19 (51%) were female. All but 1 patient had serious underlying chronic medical conditions: malignancy (n = 13, 35%), cardiovascular disease (n = 9, 24%), chronic renal insufficiency (n = 6, 16%), diabetes (n = 6, 16%), and gastrointestinal disorders (n = 4, 11%). Some patients had indwelling central venous catheters (CVCs) (n = 19, 51%) or other foreign bodies, such as prosthetic valves (n = 3), pacemakers (n = 2), peritoneal dialysis catheters (n = 2), hemodialysis catheter (n = 1), and an orthopedic external fixation device (n = 1).

The most common manifestation of infection was bacteremia (n = 22, 59%); a total of 11 patients had central line-associated bacteremia, 8 had CVC-related bacteremia, 1 had bacteremia associated with a pacemaker lead infection, and 1 had bacteremia from a hemodialysis fistula (*5*). Bacteremia occurred in 1 patient without a CVC who had undergone liver transplantation. Pneumonia occurred in 4 patients, 3 of whom had underlying pulmonary disease. Skin and soft tissue infections were reported in 3 patients, and postsurgical infections were found in 2 patients (a pacemaker pocket infection and infection at a pin exit site). Both patients with endocarditis had histories of intravenous drug abuse and had undergone previous mitral valve replacement. Both patients with peritonitis had indwelling peritoneal dialysis catheters. Other infections included single episodes of granulomatous meningitis and urinary tract infection.

Delays in identifying *M. neoaurum* were common. The median time to reporting positive cultures was 4.5 (IQR 1–10) days. Most isolates were identified as gram-positive or gram-variable coccobacilli or bacilli. In 1 case, AFB staining was delayed, leading to preliminary identification of the isolate as *Rhodococcus* sp. (*6*). In 24 cases for which the method of definitive identification was reported, investigators used chromatography (n = 6); matrix-assisted laser desorption/ionization time-of-flight mass spectrometry (n = 4); or sequencing of 16S rRNA (n = 15), the β subunit of RNA polymerase *ropB* gene (n = 1), or 65-kDa heat shock protein gene *hsp65* (n = 8). In 2 cases, a specific mycobacterial PCR was used for identification.

Of 21 isolates tested, all were susceptible to doxycycline, linezolid, and moxifloxacin according to the published report or Clinical and Laboratory Standards Institute broth microdilution interpretive criteria for RGM. Most isolates were susceptible to amikacin (17 of 18 isolates), cefoxitin (12 of 13), ciprofloxacin (15 of 16), imipenem (14 of 15), and meropenem (3 of 3) (Table) (*6**–**23*). Isolates were less reliably susceptible to TMP/SMX (10 of 15 isolates) and clarithromycin (7 of 15). Patients were treated with a variety of antimicrobial agents and regimens for a median duration of 6 (IQR 5–13) weeks; 3 patients received no antimicrobial drug therapy, and treatment details were not reported for 1 case. Combination antimicrobial drug therapy was used initially in 25 (74%) and, ultimately, 26 (76%) patients. Most (16 of 19) patients with CVC-associated bacteremia had their CVC removed. Infection management involving other medical devices often included device removal. However, several infections were treated only with antimicrobial drugs, including 1 of 2 cases of endocarditis, 1 of 2 infections associated with peritoneal dialysis catheters, 1 pacemaker pocket infection, and 1 pin tract infection. The patient with meningitis died; the long-term outcome of another patient with a urinary tract infection was not reported. Otherwise, infections in all patients were cured. One patient with CVC-related bacteremia who was treated medically had a relapse that was successfully treated by CVC removal and a second course of antimicrobial drugs. The relative risk for relapse among patients with CVC-associated infections who had their CVC removed was 0.083 (95% CI 0.0041–1.6860; p = 0.105); the number needed to treat for 1 patient to benefit was 2.9.

**Table:** Methods of determination and antimicrobial drug susceptibilities of isolates from the current case and published reports of healthcare-associated infections caused by *Mycolicibacterium* neoaurum*

Reference	Method	Antimicrobial drug susceptibility†
Case report	Broth microdilution	Amikacin, <1, S; cefoxitin, 4, S; ciprofloxacin, <0.12, S; clarithromycin, 8, R; doxycycline, <0.12, S; imipenem, 0.12, S; linezolid, <1, S; moxifloxacin, 0.06, S; tigecycline, 0.12, no interpretation; TMP/SMX, 2/38, S
(*6*)	Agar dilution	Amikacin, 0.5, S; ciprofloxacin, 0.016, S; clarithromycin, 4, I; doxycycline, 0.064, S; linezolid, 0.25, S; meropenem, 0.25, S; moxifloxacin, 0.008, S; TMP/SMX, 0.25, S
(*7*)	Etest	Amikacin, S; clarithromycin, S; TMP/SMX, S
(*8*)	Disk diffusion	Amikacin, S; cefoxitin, S; doxycycline, S; imipenem, S; TMP/SMX, S
(*9*)	Etest	Amikacin, S; cefoxitin, S; ciprofloxacin, S; clarithromycin, S; imipenem, S; linezolid, S; TMP/SMX, R
(*10*)	Broth microdilution	Amikacin, S; cefoxitin, S; ciprofloxacin, S; clarithromycin, R; doxycycline, S; imipenem, S; linezolid, S; moxifloxacin, S; TMP/SMX, S
(*11*)	Disk diffusion	Amikacin, S; cefoxitin, S; ciprofloxacin, S; clarithromycin, R; imipenem, S; TMP/SMX, R
(*12*)	Broth microdilution	Amikacin, <8, S; cefoxitin, <16, S; ciprofloxacin, <1, S; doxycycline, <1, S; imipenem, <2, S; linezolid, <1, S; moxifloxacin, <0.5, S; TMP/SMX, 1/19, S
(*13*)	Not reported	Amikacin, R; ciprofloxacin, R; imipenem, R; TMP/SMX, R
(*14*)	Not reported	Amikacin, <1, S; cefoxitin, 8, S; ciprofloxacin, <0.12, S; clarithromycin, >4, R; doxycycline, <0.25, S
(*14*)	Not reported	Amikacin, 8, S; doxycycline, 0.5, S; linezolid, 1, S
(*14*)	Broth microdilution	Amikacin, 1, S; cefoxitin, 8, S; ciprofloxacin, 0.25, S; clarithromycin, 0.25, S; imipenem, 1, S; linezolid, 2, S
(*15*)	Not reported	Amikacin, <1, S; cefoxitin, 8, S; ciprofloxacin, 0.25, S; clarithromycin, >16, R; doxycycline, 1, S; imipenem, <2, S; linezolid, 4, S; moxifloxacin, <0.25, S; TMP/SMX, 0.5/9.5, S
(*16*)	Disk diffusion	Amikacin, S; ciprofloxacin, S; doxycycline, S; imipenem, S; meropenem, S; TMP/SMX, S
(*17*)	Etest	Ciprofloxacin, S; clarithromycin, R; doxycycline, S; imipenem, S
(*18*)	Broth microdilution	Amikacin, <1.0, S; cefoxitin, 32, I; ciprofloxacin, <0.125, S; clarithromycin, 2, S; doxycycline, <0.125, S; imipenem, 2, S; linezolid, <2, S; moxifloxacin, <0.125, S; TMP/SMX, 16/304, R
(*19*)	Not reported	Amikacin, <8, S; cefoxitin, <16, S; ciprofloxacin, <1, S; clarithromycin, 1, S; doxycycline, <1, S; imipenem, <2, S; linezolid, <1, S; moxifloxacin, <0.5, S; TMP/SMX, <0.5/9.5, S
(*20*)	Etest	Cefoxitin, 2, S; ciprofloxacin, 0.6, S; clarithromycin, 0.125, S; imipenem, 0.19, S; linezolid, 1.5, S; moxifloxacin, 0.2, S; TMP/SMX, 32, R
(*21*)	Broth microdilution	Amikacin, <1, S; cefoxitin, 8, S; clarithromycin, 2, S; imipenem, <2, S; linezolid, 2, S; meropenem, 2, S; moxifloxacin, <0.25, S; TMP/SMX, 1/19, S
(*22*)	Disk diffusion	Amikacin, S; clarithromycin, R; ciprofloxacin, S; doxycycline, S
(*23*)	Etest	Imipenem, 0.12, S

*Values for each antimicrobial drug are MICs in μg/mL. Etest, bioMérieux. I, intermediate; S, sensitive; R, resistant; TMP/SMX, trimethoprim/sulfamethoxazole. †MICs were interpreted according to broth microdilution criteria in the Clinical and Laboratory Standards Institute guidelines for rapidly growing mycobacteria (*4*).

### Other Reports

In addition to the individual cases in this review, a case series of 4 patients with *M. neoaurum* bacteremia has been reported (*24*). Their median age was 54 years, and 3 were male. All 4 patients were immunocompromised (3 with hematologic malignancies, 1 with a solid tumor) but not neutropenic. Three patients were treated by catheter removal and a combination of antimicrobial drugs. In 1 case, the isolate was considered a contaminant and not treated. All infections were cured. In another report, 2 of 28 patients (both children) with cancer had bacteremia attributed to *M. neoaurum* (*25*).

## Discussion

Previous reports have described *M. neoaurum* infections as primarily affecting immunocompromised persons. However, infections that we described in our case report and literature review might be more appropriately considered healthcare-associated infections, because most patients were not immunocompromised but had medical devices or had undergone invasive procedures before infections developed. In our study, 3 patients with pulmonary infections had conditions that predisposed them to anatomic lung abnormalities and infections caused by other mycobacteria species (*26*). Furthermore, 1 patient with a skin and soft tissue infection had a history of penetrating trauma, but 2 others with this condition did not report trauma. However, injury might not have been recalled, or *M. neoaurum* inoculation might have occurred through an unrecognized skin break. In a single-center study of cutaneous nontuberculous mycobacteria infections, histories of trauma, surgical procedure, or environmental exposure to mycobacteria were common among patients; *M. neoaurum* caused 2 of 78 infections (*27*). As the population of persons with chronic medical conditions increases, more *M. neoaurum* infections will likely be recognized.

We found that 1 infection in our case series occurred in a previously healthy, 25-year-old woman who showed signs of pulmonary disease that was AFB smear positive; the infecting organism was confirmed as *M. neoaurum* by 16S rRNA sequencing (*18*). Although this finding suggests that *M. neoaurum* might cause occasional disease in healthy persons, the patient might have had an unrecognized risk factor for mycobacterial infection, such as interferon gamma receptor 1 deficiency, which would only become apparent over time (*28*). The patient responded to antimicrobial drug therapy, but her long-term outcome was not reported.

One patient in our review who had several serious medical comorbidities had rapidly progressive dementia and diagnostic imaging studies suggestive of recurrent ischemic stroke (*29*); an autopsy revealed granulomatous meningitis. Results of conventional diagnostic microbiology were uninformative, but broad-range bacterial rDNA PCR amplified a product that was 99% homologous to *M. neoaurum* DNA. However, histopathologic stains did not reveal AFB, cultures were sterile, and the patient met criteria for an alternative diagnosis of probable Creutzfeldt-Jacob disease, suggesting that PCR might have been falsely positive (*29*,*30*). Except for this case, all patients in our review were promptly cured of their infections, and no patient required intensive care or died. Thus, in contrast to other RGM species, *M. neoaurum* appears to have low virulence and is associated with limited illness and death (*26*).

*M. neoaurum* has been isolated from soil, tap water, fish, domesticated animals, and animal products (*31*–*33*). Infections are presumed to result from exposure of susceptible hosts to organisms in the environment (*34*). Nosocomial infections caused by RGM are not uncommon and are often related to contamination of medical devices, wounds, or aqueous solutions. An outbreak of *Mycobacterium mucogenicum* and *M. neoaurum* bacteremia among patients with hematologic malignancies has been reported (*7*). *M. mucogenicum* and other nontuberculous mycobacteria, but not *M. neoaurum*, were isolated from the hospital water system (water tanks, showers, wash basins). Environmental measures, such as cleaning or replacing fixtures, general cleaning, chlorinating the water supply, and minimizing stagnation, reduced but did not eliminate water contamination. After changes were made to protocols for the care of CVCs, however, no further cases were reported. Environmental samples from the hospital and home environment of a patient with pulmonary *M. neoaurum* infection were similarly analyzed (*35*). Again, other mycobacteria were isolated from these sources, but *M. neoaurum* was not identified. Therefore, additional studies will be needed to elucidate the pathogenesis and risk factors for *M. neoaurum* infection.

Delays in identifying *M. neoaurum* and obtaining susceptibility test results pose challenges to microbiologists and clinicians. The median time to initial culture positivity in this series was >4 days and as high as 10 days, which might exceed the usual incubation duration for blood cultures, leading to premature no growth determinations (*6*,*36*). Many hospital laboratories no longer routinely speciate bacteria or perform susceptibility testing, and delays in appropriate treatment might be compounded by the need to send isolates to a reference laboratory. In this case series, empirical therapy was often directed at more common RGM. In some cases, susceptibility testing was not performed, and therapy success was judged by the patient’s clinical response. The quality of care for patients with RGM infections might be improved by educating laboratory personnel regarding characteristics of less commonly identified RGM, developing protocols that promote rapid identification, and sending isolates to laboratories with specialized expertise in identification and susceptibility testing.

The optimal type and duration of antimicrobial drug therapy for *M. neoaurum* infections has not been established. *M. neoaurum* bacteria are resistant to most antituberculosis medications. In 1 study, all 46 *M. neoaurum* isolates tested were susceptible to amikacin, cefoxitin, ciprofloxacin, doxycycline, imipenem, linezolid, moxifloxacin, and TMP/SMX, but only 8% were susceptible to clarithromycin (*37*). Isolates in our case series were also susceptible to most tested antimicrobial drugs but less consistently than previously described (*37*). Of note, most reports included in our study did not describe the methodology used for testing susceptibilities or used methods that were not recommended. In particular, macrolide susceptibility might have been overestimated if prolonged incubation was not used to detect inducible macrolide resistance (*38*). Furthermore, whereas most *M. neoaurum* infections have responded well to therapy, a formal correlation between antimicrobial drug susceptibility and clinical outcomes has not been made. As in our case report, other clinicians have frequently used a combination of agents that often include a macrolide, a fluoroquinolone, or both. Although initial combination therapy might be desirable, ≈25% of patients in our case series received monotherapy or were treated by device removal alone, and 1 patient with bacteremia recovered without treatment.

The ability of RGM to cause medical device infections and subsequent need for device removal to eradicate infection has been attributed in part to RGM biofilm formation (*39*). Not all RGM produce biofilms, however, and biofilm formation by *M. neoaurum* has not been specifically investigated (*40*–*42*). Failure to remove CVCs in patients with catheter-associated bacteremia was associated with treatment failure in our case series; however, the small number of cases precludes a precise estimate of risk. Good outcomes were reported in some cases when it was not feasible to remove devices. The ideal duration of antimicrobial drug therapy is also uncertain; >4 weeks has been recommended for patients with other RGM infections (*39*). For *M. neoaurum* infections described in this report, patients with bacteremia treated with antimicrobial drugs for <4 weeks had outcomes equivalent to those receiving a longer course.

RGM treatment for patients with underlying medical disorders might be challenging because of antimicrobial drug resistance, relatively high rates of adverse events, and some medications having multiple and serious drug interactions. In our case report, the patient required ongoing treatment for ALL that included mercaptopurine, methotrexate, dexamethasone, and vincristine. The availability of a relatively large number of antimicrobial drugs to which his isolate was susceptible permitted us to continue his chemotherapy without substantial disruption. Imipenem and cilastatin do not have notable interactions with those chemotherapeutic agents. Although TMP/SMX might theoretically exacerbate myelosuppression by mercaptopurine, the combination is commonly used during ALL treatment, and we felt this treatment would be manageable. However, clarithromycin (a strong cytochrome P450 3A4 inhibitor) has potentially severe interactions with vincristine and dexamethasone and is withheld typically for a specified period before and after administration of vincristine. Ciprofloxacin used in combination with dexamethasone might increase the risk for tendinitis or tendon rupture. During ALL induction therapy, patients are usually prescribed concurrent levofloxacin (for antibacterial prophylaxis) and corticosteroids for several weeks, and we have not observed frequent or severe adverse effects (*43*). We believe that, with careful observation, benefits of this regimen exceeded risks in our patient.

In conclusion, we established that infections caused by the emerging RGM pathogen *M. neoaurum* occurred in patients with diverse demographic characteristics, but almost all cases were healthcare associated. In contrast to isolates of other RGM species, *M. neoaurum* isolates were generally susceptible to tested antimicrobial drugs; a notable exception was clarithromycin. We recommend using combination antimicrobial drug therapy and removal of infected devices, although a shorter treatment duration than is generally recommended for RGM might be effective for *M. neoaurum* infections. We found that delays in identification of isolates and susceptibility testing occurred, but outcomes of most infections were good.

Appendix Additional information for healthcare-associated infections caused by *Mycolicibacterium* *neoaurum*.
