# Supplementary material for: Healthcare-Associated Infections Caused by Mycolicibacterium neoaurum
Source: Emerg Infect Dis. 2023 Aug;29(8):1516–23. doi: 10.3201/eid2908.230007 (PMC10370869; doi:10.3201/eid2908.230007)
Supplement: Appendix — Additional information for healthcare-associated infections caused by Mycolicibacterium neoaurum. [file 23-0007-Techapp-s1.pdf]

Article DOI: https://doi.org/10.3201/eid2908.230007

EID cannot ensure accessibility for supplementary materials supplied by authors. Readers who have difficulty accessing supplementary content should contact the authors for assistance.

Healthcare-Associated Infections Caused by *Mycolicibacterium neoaurum*

Appendix

Appendix Table. Clinical characteristics and treatment of 37 reported cases of patients with *Mycolicibacterium neoaurum* infections*

No.	Age, y/sex, location	Medical condition, device duration†	Infection	Positive culture site, time to positive result	Treatment	Outcome	Reference
1	1/M, Missouri, USA	Acute lymphoblastic leukemia, subcutaneous port, 6 mo	Bacteremia, catheter-related	Port blood, 5 d	Port removed, IMI + AZI + CIP for 16 d, then TMS + CIP for 26 d	Cured	Case report
2	48/M, Michigan, USA	Acute lymphoblastic leukemia, subcutaneous port	Bacteremia, catheter-related	Blood, 5 d	Port removed, CIP + DOX for 3 mo	Cured	(*1*)
3	67/F, West Virginia, USA	Diabetes, chronic renal insufficiency, recurrent urinary tract infection, percutaneously inserted central line, 6 mo	Bacteremia, catheter-related	Catheter blood, 6 d	Central line removed, CIP + DOX for 4 wk	Not reported	(*2*)
4	41/M, Scotland, UK	Acute myeloid leukemia, neutropenia, central venous catheter	Bacteremia, catheter-associated	Catheter, peripheral blood	Central line removed, MER (duration not reported)	Cured	(*3*)
5	54/F, Queensland, Australia	Ovarian carcinoma, total parenteral nutrition, central venous catheter	Bacteremia, catheter-associated	Catheter, peripheral blood, 2–5 d	GEN + CEFO for 7 wk	Cured	(*4*)
6	31/F, Cadiz, Spain	Ulcerative colitis, peripheral intravenous catheter, 1 wk	Bacteremia, catheter-associated, exit site inflammation	Peripheral blood, 3 d	TEI for 7 d	Cured	(*5*)
7	46/M, Iowa, USA	Primary hyperparathyroidism, central venous catheter, 10 mo	Bacteremia, catheter-related	Catheter blood, 2 d	Catheter removed	Cured	(*6*)
8	48/M, Illinois, USA	Crohn’s disease, short gut syndrome, hepatitis C infection, central venous catheter	Bacteremia, catheter-related	Blood	Catheter removed, IMI + CIP, then LEV for 4 wk	Cured	(*7*)
9	17/M, New South Wales, Australia	Acute lymphoblastic leukemia, hematopoietic stem cell transplant, neutropenia, central venous catheter	Bacteremia, catheter-related, catheter exit site inflammation	Catheter blood, 2 d	Catheter removed, TCL + TOB for 3 wk	Cured	(*8*)
10	46/M, Taiwan, China	Diabetes, alcohol-related pancreatitis and hepatitis, multi-organ failure, intra-abdominal infection, prosthetic joint replacement, hepatitis B infection, cerebrovascular accident, central venous catheter	Bacteremia, catheter-associated	Catheter blood, 6 d	Catheter removed, AMI + CIP for 1 wk, then AMI + MER for 2 wk	Cured	(*9*)

No.	Age, y/sex, location	Medical condition, device duration†	Infection	Positive culture site, time to positive result	Treatment	Outcome	Reference
11	66/M, Oklahoma, USA	Diabetes, colon cancer, schizophrenia, subcutaneous port, 6 y	Bacteremia, catheter-associated	Central, peripheral blood, catheter tip, 5 d	Port removed, DOX + IMI + LEV for 4 wk, then DOX + LEV for 2 wk	Cured	(*10*)
12	53/M, Pennsylvania, USA	Hepatocellular carcinoma, hepatitis B infection, psoriasis, subcutaneous port	Bacteremia, catheter-related	Catheter blood, 7 d	CON, unspecified duration	Relapsed 3 mo, port removed, CON for 2 wk, cured	(*11*)
13	4/F, Ohio, USA	Acute myeloid leukemia, central venous catheter	Bacteremia, catheter-associated	Blood	Catheter removed, TMS for 6 wk	Cured	(*12*)
14	39/F, Ohio, USA	Aplastic anemia, central venous catheter	Bacteremia, catheter-associated	Blood	Catheter removed, CIP + CEFT + ERY + ETH + RIF + CEFT for 6 wk	Cured	(*12*)
15	68/M, Illinois, USA	Diabetes, recurrent small bowl obstruction, parenteral nutrition, recurrent urinary tract infection, central venous catheter, 3 y	Bacteremia, catheter-related, pneumonia	Blood, catheter tip, 8 d	Catheter removed, CEFO + CIP + DOX for 6 wk, then CIP + DOX for 3 wk	Cured	(*13*)
16	1/F, Michigan, USA	Liver transplant	Bacteremia	Peripheral blood, 6 d	None	Cured	(*14*)
17	2/F, Michigan, USA	Neuroblastoma, neutropenia, central venous catheter, 5 mo	Bacteremia, catheter-associated	Blood, 4 d	Catheter removed, AMI + CLA + MER for 7 d, then CIP + LIN for 14 d	Cured	(*14*)
18	3/M, Michigan, USA	Rhabdomyosarcoma, neutropenia, central venous catheter, 7 mo	Blood, catheter-associated	Blood, 1 d	Catheter removed, AMI + CLA + LEV for 12 d, then CLA + LEV for 4 wk	Cured	(*14*)
19	59/F, Michigan, USA	Colon cancer, colectomy, short gut syndrome, percutaneously inserted central line, 1 mo	Blood, catheter-associated	Blood, 2 d	Catheter removed, CEFO for 2 wk, then CLA + ETHA + LEV for 4 wk	Cured	(*14*)
20	9/F, Hong Kong, China	Acute lymphoblastic leukemia, central venous catheter	Bacteremia, catheter-related	Catheter blood, 3 d	Catheter removed, CEFZ + AMI for 3 wk	Cured	(*15*)
21	40/F, Manitoba, Canada	Diabetes, chronic renal insufficiency, hepatitis C infection, hemodialysis fistula, 9 mo	Bacteremia	Blood, hemodialysis fistula, peripheral, excised graft	Excision hemodialysis fistula, CIP + DOX for 4 wk	Cured	(*16*)
22	80/F, England, UK	Heart block, pacemaker, 2 y	Bacteremia, pacemaker-associated infection	Blood, pacemaker leads, 4 d	Pacemaker removed, AMI + IMI for 23 d, then CIP + DOX + LIN for 1 mo, then CIP + DOX for 2 mo	Cured	(*17*)
23	30/M, Connecticut, USA	Intravenous drug abuse, endocarditis, prosthetic mitral valve replacement, 1 y	Prosthetic valve endocarditis, brain abscess	Blood, 10 d	AZI + MOX + TOB for 39 d, then AZI + ETH + MOX, duration not reported	Cured	(*18*)
24	36/M, Ohio, USA	Intravenous drug abuse, prosthetic mitral valve replacement, 1 y	Prosthetic valve endocarditis	Blood, mitral valve	Mitral valve replacement, CIP + IMI + TMS for 6 wk, then CIP + TMS suppression	Cured	(*12*)
25	63/F, Ontario, Canada	Cerebrovascular accident, rheumatoid arthritis, hypertension, corticosteroid use, tobacco smoker	Granulomatous meningitis	Broad range PCR, brain tissue	None	Death	(*19*)
26	17/M, Moravia, Czech Republic	Hodgkin lymphoma, malignant pleural effusion, neutropenia	Pneumonia	Sputum	CIP for 3 mo	Cured	(*20*)

No.	Age, y/sex, location	Medical condition, device duration†	Infection	Positive culture site, time to positive result	Treatment	Outcome	Reference
27	25/F, Chungcheong, South Korea	None	Pneumonia	Bronchoalveolar lavage fluid, 4 d	CLA for 4 mo	Cured	(*21*)
28	59/M, New South Wales, Australia	Emphysema, tobacco smoker, tuberculosis	Pneumonia	Sputum	MOX + RIF + TMP/SMX for 6 mo	Cured	(*22*)
29	67/F, Wisconsin, USA	Asthma, corticosteroid use, gastroesophageal reflux, dental caries, tobacco smoker	Pneumonia	Lung biopsy	AMI + CLA for 6 mo	Cured	(*23*)
30	63/F, Gran Canaria, Spain	Heart block, pacemaker, 11 d, hypertension, hypothyroid, breast cancer	Surgical wound infection, pacemaker pocket	Wound exudate, 10 d	LEV for 2 wk, then DOX + LEV for 4 wk	Cured	(*24*)
31	14/F, Chiba, Japan	Orthopedic surgery, external fixator, 18 d	Pin exit site infection	Pin tract exudate, 3 d	CIP + MIN, then MIN + TMS for 6 mo	Cured	(*25*)
32	76/M, England, UK	Chronic renal insufficiency, hypertension, peripheral vascular disease	Soft tissue infection	PCR, skin biopsy	CIP + DOX for 6 mo	Cured	(*26*)
33	58/M, Hong Kong, China	Penetrating hand injury, sea water exposure, hypothyroidism	Soft tissue infection	Tissue biopsy	Soft tissue debridement, CIP + DOX for 12 wk	Cured	(*27*)
34	53/F, New South Wales, Australia	Mitral valve replacement, hypertension	Soft tissue infection	PCR, skin biopsy	MOX + ROX for 4 mo	Cured	(*28*)
35	45/F, New South Wales, Australia	Chronic renal insufficiency, peritoneal dialysis	Peritonitis	Peritoneal fluid	GEN + VAN, then AMI + PIP, no duration given	Cured	(*29*)
36	54/M, Ohio, USA	Diabetes, hypertension, chronic renal insufficiency, continuous ambulatory peritoneal dialysis	Peritonitis	Peritoneal fluid	Dialysis catheter removed, AMI + CIP + RIF for 4 wk, then CIP + RIF for 3 mo	Cured	(*30*)
37	63/F, Sardinia, Italy	Chronic renal insufficiency, nephrolithiasis, recurrent urinary tract infection	Urinary tract infection	Urine, 6 d	Not reported	Cured	(*31*)

*AMI, amikacin; AZI, azithromycin; CEFO, cefoxitin; CEFT, ceftriaxone; CEFZ, ceftazidime; CIP, ciprofloxacin; CLA, clarithromycin; CON, contezolid; DOX, doxycycline; ERY, erythromycin; ETHA, ethambutol; GEN, gentamicin; IMI, imipenem; LEV, levofloxacin; LIN, linezolid; MER, meropenem; MIN, minocycline; MOX, moxifloxacin; PIP, piperacillin; RIF, rifampin; ROX, roxithromycin; TCL, ticarcillin-clavulanate; TEI, teicoplanin; TMS, trimethoprim-sulfamethoxazole; TOB, tobramycin; VAN, vancomycin.
†Medical conditions included the use of medical devices and duration of use, if reported.

References

1. Alhusseini M, Miceli MH, Chandrasekar P, Revankar S. Catheter-related bloodstream infection due to *Mycobacterium neoaurum* in a patient with acute leukemia. Leuk Lymphoma. 2014;55:1933–4. PubMed https://doi.org/10.3109/10428194.2013.858153

2. Awadh H, Mansour M, Shorman M. Bacteremia with an unusual pathogen: *Mycobacterium neoaurum.* Case Rep Infect Dis. 2016;2016:5167874. PubMed https://doi.org/10.1155/2016/5167874

3. Baird SF, Taori SK, Dave J, Willocks LJ, Roddie H, Hanson M. Cluster of nontuberculous mycobacteraemia associated with water supply in a haemato-oncology unit. J Hosp Infect. 2011;79:339–43. PubMed https://doi.org/10.1016/j.jhin.2011.07.006

4. Davison MB, McCormack JG, Blacklock ZM, Dawson DJ, Tilse MH, Crimmins FB. Bacteremia caused by *Mycobacterium neoaurum.* J Clin Microbiol. 1988;26:762–4. PubMed https://doi.org/10.1128/jcm.26.4.762-764.1988

5. Rubia MF, Chozas N, García-Martos P, Reyes F. *Mycobacterium neoaurum* bacteremia in an immunodepressed patient [in Spanish] Enferm Infecc Microbiol Clin. 2009;27:58–9. PubMed https://doi.org/10.1016/j.eimc.2008.02.003

6. George SL, Schlesinger LS. *Mycobacterium neoaurum*—an unusual cause of infection of vascular catheters: case report and review. Clin Infect Dis. 1999;28:682–3. PubMed https://doi.org/10.1086/517216

7. Hawkins C, Qi C, Warren J, Stosor V. Catheter-related bloodstream infections caused by rapidly growing nontuberculous mycobacteria: a case series including rare species. Diagn Microbiol Infect Dis. 2008;61:187–91. PubMed https://doi.org/10.1016/j.diagmicrobio.2008.01.004

8. Holland DJ, Chen SC, Chew WW, Gilbert GL. *Mycobacterium neoaurum* infection of a Hickman catheter in an immunosuppressed patient. Clin Infect Dis. 1994;18:1002–3. PubMed https://doi.org/10.1093/clinids/18.6.1002

9. Lai CC, Tan CK, Chen CC, Hsueh PR. *Mycobacterium neoaurum* infection in a patient with renal failure. Int J Infect Dis. 2009;13:e276–8. PubMed https://doi.org/10.1016/j.ijid.2008.11.001

10. Moseley JE Jr, Thind SK. *Mycobacterium neoaurum* bloodstream infection associated with a totally implanted subclavian port in an adult with diabetes and history of colon cancer. Case Rep Infect Dis. 2020;2020:8878069. PubMed https://doi.org/10.1155/2020/8878069

11. Pang L, Chen Z, Xu D, Cheng W. Case report: *Mycobacterium neoaurum* infection during ICI therapy in a hepatocellular carcinoma patient with psoriasis. Front Immunol. 2022;13:972302. PubMed https://doi.org/10.3389/fimmu.2022.972302

12. van Duin D, Goldfarb J, Schmitt SK, Tomford JW, Tuohy MJ, Hall GS. Nontuberculous mycobacterial blood stream and cardiac infections in patients without HIV infection. Diagn Microbiol Infect Dis. 2010;67:286–90. PubMed https://doi.org/10.1016/j.diagmicrobio.2010.02.006

13. Walayat S, Awwal T, Roy M, Ahmad S. *Mycobacterium neoaurum* line-related bacteremia with pulmonary involvement: case report and review of literature. IDCases. 2018;11:88–90. PubMed https://doi.org/10.1016/j.idcr.2018.01.004

14. Washer LL, Riddell J 4th, Rider J, Chenoweth CE. *Mycobacterium neoaurum* bloodstream infection: report of 4 cases and review of the literature. Clin Infect Dis. 2007;45:e10–3. PubMed https://doi.org/10.1086/518891

15. Woo PC, Tsoi HW, Leung KW, Lum PN, Leung AS, Ma CH, et al. Identification of *Mycobacterium neoaurum* isolated from a neutropenic patient with catheter-related bacteremia by 16S rRNA sequencing. J Clin Microbiol. 2000;38:3515–7. PubMed https://doi.org/10.1128/JCM.38.9.3515-3517.2000

16. Becker ML, Suchak AA, Wolfe JN, Zarychanski R, Kabani A, Nicolle LE. *Mycobacterium neoaurum* bacteremia in a hemodialysis patient. Can J Infect Dis. 2003;14:45–8. PubMed https://doi.org/10.1155/2003/840103

17. Hayton ER, Koch O, Scarborough M, Sabharwal N, Drobniewski F, Bowler ICJW. Rapidly growing mycobacteria as emerging pathogens in bloodstream and device-related infection: a case of pacemaker infection with *Mycobacterium neoaurum*. JMM Case Rep. 2015;2:1–3. https://doi.org/10.1099/jmmcr.0.000054

18. Kumar A, Pazhayattil GS, Das A, Conte HA. *Mycobacterium neoaurum* causing prosthetic valve endocarditis: a case report and review of the literature. Braz J Infect Dis. 2014;18:235–7. PubMed https://doi.org/10.1016/j.bjid.2013.05.012

19. Heckman GA, Hawkins C, Morris A, Burrows LL, Bergeron C. Rapidly progressive dementia due to *Mycobacterium neoaurum* meningoencephalitis. Emerg Infect Dis. 2004;10:924–7. PubMed https://doi.org/10.3201/eid1005.030711

20. Kaevska M, Sterba J, Svobodova J, Pavlik I. *Mycobacterium avium* subsp. *avium* and *Mycobacterium neoaurum* detection in an immunocompromised patient. Epidemiol Infect. 2014;142:882–5. PubMed https://doi.org/10.1017/S0950268813001660

21. Kim CK, Choi SI, Jeon BR, Lee YW, Lee YK, Shin HB. Pulmonary infection caused by *Mycobacterium neoaurum*: the first case in Korea. Ann Lab Med. 2014;34:243–6. PubMed https://doi.org/10.3343/alm.2014.34.3.243

22. Josan E, Singh S, Bark C, Infeld M. *Mycobacterium neoaurum* with isolated cavitary pulmonary infection. Chest. 2020;158:A370. https://doi.org/10.1016/j.chest.2020.08.365

23. Morimoto Y, Chan ED, Heifets L, Routes JM. Pulmonary infection with *Mycobacterium neoaurum* identified by 16S ribosomal DNA sequence. J Infect. 2007;54:e227–31. PubMed https://doi.org/10.1016/j.jinf.2006.12.010

24. Bastón-Paz N, Bolaños-Rivero M, Hernández-Cabrera M, Martín-Sánchez AM. Pacemaker infection with *Mycobacterium neoaurum* [in Spanish]. Rev Esp Quimioter. 2018;31:379–82. PubMed

25. Kusano T, Fukasawa C, Yamamoto S, Shiratori E, Murata S, Takaki A, et al. Pin tract infection caused by *Mycobacterium neoaurum* in a 14-year-old child: a case report. J Infect Chemother. 2021;27:1244–7. PubMed https://doi.org/10.1016/j.jiac.2021.03.005

26. Chadha M, Arias M, Maxwell-Scott H, Creamer D, Boissiere J, Sioletic S, et al. *Mycobacterium neoaurum* as an unusual cause of skin and soft tissue infection. Int J Dermatol. 2023;62:e45–7. PubMed https://doi.org/10.1111/ijd.16424

27. Omoruyi OJ, Ip WY, To KK. Hand infection due to *Mycobacterium neoaurum.* J Hand Surg Eur Vol. 2012;37:574–5. PubMed https://doi.org/10.1177/1753193412442296

28. Martin LK, Lawrence R, Kossard S, Murrell DF. Cutaneous *Mycobacterium neoaurum* infection causing scarring alopecia in an immunocompetent host. Br J Dermatol. 2007;157:204–6. PubMed https://doi.org/10.1111/j.1365-2133.2007.07953.x

29. Jiang SH, Roberts DM, Clayton PA, Jardine M. Nontuberculous mycobacterial PD peritonitis in Australia. Int Urol Nephrol. 2013;45:1423–8. PubMed https://doi.org/10.1007/s11255-012-0328-4

30. McNally CF, Mangino JE. *Mycobacterium neoaurum*: a case report and review of the literature. Infect Dis Clin Pract. 2000;9:273–5. https://doi.org/10.1097/00019048-200009060-00013

31. Zanetti S, Faedda R, Fadda G, Dupré I, Molicotti P, Ortu S, et al. Isolation and identification of *Mycobacterium neoaurum* from a patient with urinary infection. New Microbiol. 2001;24:189–92. PubMed
